# Telehealth methods to deliver multifactorial dietary interventions in adults with chronic disease: a systematic review protocol

**DOI:** 10.1186/s13643-015-0170-8

**Published:** 2015-12-22

**Authors:** Jaimon T. Kelly, Dianne P. Reidlinger, Tammy C. Hoffmann, Katrina L. Campbell

**Affiliations:** Faculty of Health Science and Medicine, Bond University, Gold Coast, Australia; Centre for Research in Evidence Based Practice, Faculty of Health Sciences and Medicine, Bond University, Gold Coast, Australia; Nutrition and Dietetics Department, Princess Alexandra Hospital, Brisbane, Australia

**Keywords:** Telehealth, Dietary, Diet quality, Adults, Chronic disease, Behaviour change, Lifestyle change, Technology

## Abstract

**Background:**

The long-term management of chronic diseases requires adoption of complex dietary recommendations, which can be facilitated by regular coaching to support sustained behaviour change. Telehealth interventions can overcome patient-centred barriers to accessing face-to-face programs and provide feasible delivery methods, ubiquitous and accessible regardless of geographic location. The protocol for this systematic review explains the methods that will be utilised to answer the review question of whether telehealth interventions are effective at promoting change in dietary intake and improving diet quality in people with chronic disease.

**Methods/design:**

A structured search of Medline, EMBASE, CINAHL, and PsychINFO, from their inception, will be conducted. We will consider randomised controlled trials which evaluate complex dietary interventions in adults with chronic disease. Studies must provide diet education in an intervention longer than 4 weeks in duration, and at least half of the intervention contact must be delivered via telehealth. Comparisons will be made against usual care or a non-telehealth intervention. The primary outcome of interest is dietary change with secondary outcomes relating to clinical markers pre-specified in the methodology. The process for selecting studies, extracting data, and resolving conflicts will follow a set protocol. Two authors will independently appraise the studies and extract the data, using specified methods. Meta-analyses will be conducted where appropriate, with parameters for determining statistical heterogeneity pre-specified. The GRADE tool will be used for determining the quality of evidence for analysed outcomes.

**Discussion:**

To date, there has been a considerable variability in the strategies used to deliver dietary education, and the overall effectiveness of telehealth dietary interventions for facilitating dietary change has not been reviewed systematically in adults with chronic disease. A systematic synthesis of telehealth strategies will inform the development of evidence-based telehealth programs that can be tailored to deliver dietary interventions specific to chronic disease conditions.

**Systematic review registration:**

PROSPERO CRD42015026398

**Electronic supplementary material:**

The online version of this article (doi:10.1186/s13643-015-0170-8) contains supplementary material, which is available to authorized users.

## Background

Non-pharmacological treatment methods are commonly used for people with chronic diseases [1], and many of these require lifetime adherence to dietary recommendations. Telehealth technologies can provide education and self-management support to facilitate and sustain lifestyle changes. Such interventions (which include telephone coaching, the Internet, mobile phone applications) could have advantages over traditional face-to-face models of care [2], and utilisation of them may assist with achieving dietary behaviour change [2–4].

### Description of the condition

Chronic disease is the leading cause of ill health, accounting for 68 % of all deaths worldwide [5], in some countries contributing to over 90 % of all deaths [6]. Chronic diseases are those with multifactorial aetiologies, and once diagnosed, are with the individual for life without a specific cure. Many chronic diseases are diet-related, specifically obesity, heart disease, diabetes, hypertension, stroke, and kidney disease as previously defined in a systematic review [7]. These pose a significant challenge to the health system, in terms of costs and cause of death and disability, which tends to be related to cardiovascular disease (CVD) as either the primary or co-morbid condition [8]. Self-management and the adoption of a healthy lifestyle, such as through diet, physical activity, and other health-related behaviours (e.g. smoking cessation), are considered essential for the management of these diet-related chronic diseases [9, 10].

Telephone-delivered interventions for smoking cessation [11] show improvements in quit rates, and physical activity [2] interventions delivered at least 50 % by telephone show increases in all measures of physical activity, which is also sustained following the conclusion of interventions. However, the findings from studies aimed at increasing adherence to specific dietary recommendations in chronic disease lack consistent findings in controlled studies [12].

### Description of the intervention

According to the World Health Organisation [13], the definition of telehealth is encompassed by the definition of telemedicine, which refers to the delivery of healthcare services at a distance, using information and communication technologies to exchange health information. A distinguishing characteristic of telemedicine is that it is restricted to healthcare delivery by physicians only, whereas telehealth services are provided by any health professional and can include either synchronous (i.e. same time, different location) and asynchronous (i.e. different time, different location) patient education, counselling, and remote monitoring [13]. A telehealth lifestyle intervention may involve the provision of lifestyle education or advice to an individual or group of individuals remotely via the telephone [14], computer, and the Internet [15–17], videos [18], email [19], and/or mobile phone applications including text, photo messages (short message service (SMS), or multimedia message service (MMS)) [20, 21].

### How the intervention might work

For lifestyle interventions to achieve long-term behaviour change, an intensive approach which involves frequent engagement and ongoing monitoring is recommended [1, 9, 12]. This is particularly true for changes that require the long-term maintenance of dietary strategies, which require a high degree of self-management and are notorious for poor compliance and high participant burden [22]. To improve compliance, regular contact with treating clinicians and an emphasis on self-monitoring have been suggested as central to the success of a complex dietary intervention [23, 24]. Interventions which use telehealth strategies offer expedient and feasible ways to provide the recommended support to individuals to facilitate behaviour change. For example, individuals who have limited time to attend face-to-face education could access an education program from the comfort of their own home at a time of their choosing [25]. An advantage of telehealth interventions is that educational content can be provided live (synchronous) between patient and health professional, or through text messages, emails, and Internet outlets (asynchronous and mobile health), thus overcoming some of the common barriers to face-to-face care.

### Why it is important to do this review

Technology to deliver health-related interventions have been used for over 25 years with mixed results, ranging from no effect at all to significant improvements in health outcomes [26]. Despite a range of telehealth methods for the management of chronic disease [2, 8, 27–31], as well as CVD risk behaviours [8], the effectiveness of telehealth interventions to facilitate dietary change has not been systematically synthesised. A recent systematic review demonstrated that telephone coaching is feasible for establishing effective behavioural change for physical activity and/or dietary interventions [2]. However, only two dietary studies met the inclusion criteria for this review, and studies were not specific to chronic disease. A recent systematic review demonstrated that telephone coaching is feasible for establishing effective behavioural change for physical activity and/or dietary interventions [2]. However, only two dietary studies met the inclusion criteria for this review, and studies were not specific to chronic disease. Another (Cochrane) review investigated the effectiveness of different interventions, in promoting adherence to dietary recommendations [12]. However in this review, although some included studies may have used telehealth as a component of the intervention, they did not evaluate the effectiveness of telehealth interventions specifically and did not compare to usual care (defined in its broadest sense, and which could include non-telehealth-delivered dietary advice from a health professional or no dietary education at all).

Despite a number of previous systematic reviews covering different combinations of telehealth and/or population groups (healthy and chronic disease), these reviews have not specifically evaluated interventions that attempt to change dietary patterns (i.e. multiple food groups or nutrients) which represent the dietary management of chronic disease [32].

Recent technology-based trials educating to the dietary guidelines (via the telephone) [14] and the DASH diet (via the Internet) [33] showed significant improvements in measures of dietary intake (such as fruit and vegetable consumption) for strategies using technology compared to those using more traditional strategies. Such evidence is promising for future healthcare as it may inform the development of evidence-based telehealth programs, which can be tailored to specific chronic disease conditions and may provide policy makers with more efficient options for funding programs for chronic disease management.

Although promising, to establish the effectiveness of telehealth interventions and inform future programs, telehealth interventions need to be systematically evaluated against these traditional educational strategies and to standard care alone to prompt changes in healthcare policy that have been long suggested to deal with lacking compliance to lifestyle recommendations and other barriers in current chronic disease healthcare [10].

However, there is no existing or registered systematic review that has sought to assess the overall effectiveness of telehealth dietary interventions for facilitating complex dietary change in adults with chronic disease to date.

### Objective

The objective of this review is to assess the effectiveness of telehealth as a strategy to deliver complex dietary interventions in adults with chronic disease.

## Methods/design

### Eligibility criteria

#### Study designs

Eligible designs will be randomised controlled trials (RCTs), cluster RCTs, and quasi-RCTs (RCTs using pseudo-randomisation). Trials which use crossover designs can introduce potential carry-over effects given the nature of dietary interventions to establish behaviour change; therefore, we will only include data from the first period of each intervention arm [34].

#### Participants

Adult participants (>18 years of age) with an established diet-related chronic disease which we define as obesity (BMI ≥30 kg/m^2^), diabetes mellitus, established heart disease, hypertension, stroke, and kidney disease. These diet-related chronic diseases have been previously defined in a systematic review of dietary interventions [12]. We will review studies that report on a mixed sample, however, will only include participants with a chronic disease as defined above, and provided their results are reported separately to participants that do not meet our inclusion criteria.

#### Interventions

Eligible interventions will be those that provide a multifactorial dietary intervention using at least one telehealth strategy with a duration of at least 4 weeks. We define a multifactorial dietary intervention as targeting more than a singular nutrient and/or food group. Multifactorial dietary interventions include those aimed at overall dietary patterns, such as dietary guidelines [35, 36], the Mediterranean diet [37], and/or the DASH diet or those educating on two or more dietary components (nutrients and/or food groups) simultaneously. Studies that target two or more diet changes within the same nutrient (e.g. manipulation of categories of fatty acids) will be excluded as the dietary components only relate to one nutrient, and thus are not multifactorial.

Interventions that use either a single or multifactorial telehealth strategy will be eligible. Interventions that use both telehealth and non-telehealth strategies (e.g. face-to-face, group counselling) to provide dietary education will be eligible as long as at least 50 % of the total contact hours and/or the total number of interaction contacts with participants are delivered via telehealth methods. An example of an interaction is a text message, a phone call, log-on to a webpage, or a contact session with an intervention provider. Eligible interventions will be delivered by a qualified healthcare professional (such as a nurse, dietitian, or physician).

#### Comparators

The comparison group may have received usual care (as defined by trial authors); dietary education in a face-to-face or group-based environment with no telehealth component, or via a method in which less than 50 % of the intervention is delivered via telehealth; or a non-dietary focussed intervention.

#### Outcomes

We will only include studies that report at least two measures of dietary intake: at baseline and at least 4 weeks later at follow-up.

Primary outcomes:

- Dietary intake: any objective measure of dietary intake (such as diet quality score, servings of fruits and vegetables, and nutrient intake)

Although surrogate outcomes such as dietary intake cannot reliably predict clinical outcomes (e.g. mortality), dietary intake is the first line management strategy in chronic diseases [10]. It is clinically relevant to chronic disease as it may improve clinical outcomes and is a practical policy tool to inform the development of evidence-based telehealth programs, particularly for the chronic diseases listed above. Dietary intake is measured in a variety of units, and we have chosen not to restrict our primary outcome by unit of measure. Furthermore, this review may identify which outcome measures of dietary intake are stronger as surrogate markers of our secondary clinical outcomes.

Secondary outcomes:

- All-cause mortality;
- Cardiovascular mortality;
- Hospitalisations (total and those related to chronic disease);
- Any clinical marker of chronic disease progression, such as blood pressure, estimated glomerular filtration rate (eGFR), HbA1c, weight, waist circumference, and blood lipid profiles.

#### Setting

Studies will be eligible if the intervention is conducted in ambulatory or community settings (e.g. patients can be recruited during a hospital admission, but the telehealth intervention is delivered post-discharge). Studies that are solely conducted in hospitals or controlled conditions (e.g. where food and/or beverages are provided in full or partial) will not be eligible.

#### Language

No language restriction will be in our search strategy. We will attempt to translate potentially eligible non-English articles via Google Translate or by a native speaker of the language of the article. In the event that an article is eligible but unable to be satisfactorily translated, we will present the title and author details in a supplementary appendix.

### Search methods

#### Electronic searches

A multi-step search approach will be undertaken to retrieve relevant studies. The following databases will be searched using a variety of subject headings, free text terms, and synonyms relevant to the review in consultation with an experienced search trials co-ordinator (see Additional file 1):

- MEDLINE (via OVID);
- CINAHL (via EBSCO);
- PsychINFO (via OVID); and
- EMBASE.
- Searches of the International Clinical Trials Register (ICTRP) Search Portal and ClinicalTrials.gov will be conducted to identify trials that are ongoing.

We will perform forward and backward citation searching of eligible studies. We will attempt to locate unpublished trials by contacting investigators known to be involved in previous studies that have not yet been published and by contacting published authors in the field of telehealth research and asking if they are aware of ongoing and unpublished trials.

Finally, we will perform a search for relevant theses and dissertations (via ProQuest) and conference abstracts (such as the annual meetings for the American Telemedicine Association, the International Conference on Health Informatics, and the Australasian Telehealth Society).

#### Selection of studies

All search results will be merged into reference management software EndNote, and duplicate records of the same report will be removed using the Centre for Research and Evidence Based Practice Systematic Review Assistant ‘deduplication tool’ [38]. Two review authors (JK and KC) will independently assess the eligibility of studies by screening titles and abstracts for potential inclusion according to predefined selection criteria. Studies judged to be potentially relevant will be retrieved in full text for further analysis. Any disagreements in judgement will be resolved by discussion to reach a consensus or if this is not possible, with a third review author (DR) until a consensus is reached. If further information about the study is required in order to make a decision about its eligibility, an attempt will be made to contact the study corresponding author(s). If a response is not received after three reminders are sent and/or after attempting to contact another author of the paper with no response, then the study will be excluded.

### Data collection and analysis

#### Data extraction and management

Two independent authors will extract the data independently (JK and KC). Data will be extracted from all published reports of included studies using a data extraction form which will be piloted following adaptation from the Cochrane Effective Practice and Organisation of Care Group tool [39]. For all included studies, we will extract relevant data including all details about the intervention (following the components outlined in the Template for Intervention Description and Replication (TIDieR) checklist) [40], the participants (chronic disease state, age, and gender), attrition, and all our pre-specified primary and secondary outcome data that are reported at baseline and follow-up. All extracted data will be transferred into Revman software (JK) and will be checked for accuracy (KC) prior to meta-analysis.

#### Assessing the risk of bias

Risk of bias will be assessed by two review authors (JK & KC) using the Cochrane risk of bias tool addressing the following elements that potentially affect risk of bias:

- Random sequence generation;
- Allocation concealment;
- Blinding—clients, providers, and outcome assessors;
- Incomplete outcome data;
- Selective reporting;
- Other bias.

Any disagreements in judgement will be resolved by discussion to reach a consensus or with a third review author until consensus is reached. We will tabulate and narratively describe the risk of bias in the included studies. For each study, we will categorise the risk of bias elements as low, unclear, or high risk. The effect that studies with a high risk of bias may have on the body of evidence will be explored in sensitivity analyses described below. We will consider the risk of bias for each outcome when grading the quality of the evidence.

#### Data analysis

The overall treatment effect for primary and secondary outcomes will be calculated from each trial included. The treatment effect will be calculated, where possible, as the difference between the intervention and comparison’s change from baseline to the end of follow-up for each of the measured outcomes. Variance will be calculated for each treatment effect, either derived from the standard deviation or standard error from the difference between baseline and follow-up or from confidence intervals when these are not available [34].

#### Measures of treatment effect

Where the studies included have reported interventions and outcomes which are sufficiently homogeneous, and if there is sufficient information retrieved from the studies, quantitative data will be pooled into Revman (Version 5.3) for meta-analysis using the DerSimonian and Laird random-effects model [41]. Fixed-effect model will also be used to ensure robustness and susceptibility to outliers. Effect sizes (for continuous data; e.g. diet intake, biomarkers, blood pressure, and weight) will be calculated as mean differences (MD) or as the standardised mean difference (SMD) if different scales have been used, and their 95 % confidence intervals will be calculated to measure the treatment effect. The ratio of means (RoMs) is an alternate method for data pooling [42, 43] and will be alternatively used if the SMD cannot be calculated from the outcome measures extracted from the included studies. We will convert other forms of data into MD, SMD, or RoMs and calculate confidence intervals as required. Dichotomous outcome data (e.g. death, hospitalisations, and progression to renal replacement therapy) will be expressed as risk ratios (RR) with 95 % confidence intervals. We will convert other relevant binary data into an RR value. In the event of missing data, we will attempt to impute missing standard deviations or standard errors using data from other similar studies in the review utilising similar methods and sample sizes, as recommended [44].

### Assessment of heterogeneity

We will assess the inconsistencies between studies using the *I*^2^ statistic and describe the percentage of variability in effect. Heterogeneity will be considered substantial if the *I*^2^ statistic is >50 %. We will use Egger’s plot to assess and report on potential publication bias. We will consider a sensitivity analysis to explore statistical heterogeneity. The sensitivity analysis will be considered if the results of an individual study appear to be heterogeneous with the results of other included studies; repeat the analysis excluding unpublished studies; repeat the analysis excluding high risk of bias studies (method of randomisation, allocation concealment, blinding of outcome assessor, incomplete outcome data, selective reporting, other bias); and repeat the analysis excluding any long duration studies or large studies in order to establish how much they influence effect sizes.

#### Subgroup analyses

Depending on the included studies, we will conduct subgroup analysis to explore expected sources of clinical heterogeneity. Subgroup analyses will be considered on studies conducted in those with different chronic health conditions (e.g. diabetes, established heart disease, chronic kidney disease, and hypertension); studies using different telehealth strategies (e.g. Internet or mobile phone); studies with multiple telehealth modes of delivery (e.g. Internet and telephone) versus single mode; studies greater than 6 months versus less than 6 months duration; studies where dietary education is provided in the comparison group; studies targeting specified food groups or multiple nutrients (e.g. modifying sodium, fat, fruit and/or vegetables interventions) versus dietary patterns; and studies where dietary intervention is either the sole focus of an intervention versus as part of a complex multicomponent intervention (i.e. diet and exercise).

#### Presenting and reporting of results

This protocol follows the Preferred Reporting Items for Systematic Review and Meta-Analysis Protocols (PRISMA-P) 2015 Statement [45] (see Additional file 2) . We will present the results of this review according to the Preferred Reporting Items for Systematic Reviews and Meta-Analyses (PRISMA) guidelines, using a flow diagram to report the identification and selection of studies, and assign a grading to the evidence using the GRADE tool. The relevant outcomes and characteristics of each study will be reported in summary tables. Where statistical pooling is not possible, the findings will be alternatively presented in a narrative form including tables and figures to aid in data presentation where appropriate. We will follow the Cochrane handbook guidelines for narrative synthesis, whereby grouping similar studies under headings (e.g. similar telehealth methods, dietary education, comparator studied (usual care and other dietary education delivery methods), chronic disease, dietary outcome measures) and report the direction, size and consistency of effect, and the overall quality of the body of evidence. For trials with more than one time point measurement for outcomes, we will only report results extracted from the furthest time points of the intervention.

#### Interpretation of findings

The results of the review will be discussed in the context of the quality of the evidence (GRADE), the limitations of the review, and the strengths of findings as well as their implications for current practice, future directions, and overall public health. To interpret the overall effectiveness of telehealth interventions and allow policy makers to effectively determine their efficacy at facilitating multifactorial dietary changes, we will also discuss the results in the context of the comparator studied (usual care and other dietary education delivery methods) as necessary given the retrieved body of evidence.

## Discussion

This protocol for a systematic review of available evidence will establish whether telehealth is an effective strategy to deliver multifactorial dietary interventions in adults with chronic disease, which has not been previously evaluated or reviewed systematically. If telehealth is found to be effective in establishing multifactorial dietary change, this may inform a change in current clinical and public health practice by restructuring funding and resources for future chronic disease dietary management in healthcare. Furthermore, the primary results of this review and any long-term adverse consequences found by the review may be used to inform the development of evidence-based telehealth programs, best practice guidelines, and recommendations for future telehealth intervention delivery, which can be tailored to specific chronic disease conditions. This review will also identify any apparent gaps in the body of literature and highlight priorities for future research in the area.

## Additional files

Additional file 1:
**Search strategy.** Additional file 1 presents the MEDLINE search strategy which will be used to identify potential studies. (PDF 153 kb) Additional file 2:
**PRISMA-P checklist.** Additional file 2 presents the PRISMA-P checklist. (PDF 152 kb)

## Abbreviations

BMI: body mass index
CVD: cardiovascular disease
DASH: dietary approaches to stop hypertension
eGFR: estimated glomerular filtration rate
GRADE: grading of recommendations assessment, development and evaluation
HbA1c: glycated haemoglobin
ICTRP: International Clinical Trials Register
MD: mean difference
MMS: multimedia message service
PRISMA: Preferred Reporting Items for Systematic Review and Meta-Analysis
PRISMA-P: Preferred Reporting Items for Systematic Review and Meta-Analysis Protocols
Quasi-RCT: quasi-experimental controlled trial
RCT: randomised controlled trial
RoMs: ratio of means
RR: relative risk
SD: standard deviation
SMD: standardised mean difference
SMS: short message service
TIDieR: template for intervention description and replication
