# Supplementary material for: Telehealth methods to deliver multifactorial dietary interventions in adults with chronic disease: a systematic review protocol
Source: Syst Rev. 2015 Dec 22;4:185. doi: 10.1186/s13643-015-0170-8 (PMC4689045; doi:10.1186/s13643-015-0170-8)
Supplement: Additional file 1: — Search strategy. Additional file 1 presents the MEDLINE search strategy which will be used to identify potential studies. (PDF 153 kb) [file 13643_2015_170_MOESM1_ESM.pdf]

Additional file 1: Search strategy

MEDLINE and PsychINFO (via OVID)

1. exp Telecommunications/
2. exp Internet/
3. exp Computers/ or exp Computers, Handheld/
4. Telecommunications.tw.
5. Internet.tw.
6. Telehealth.tw.
7. Telemedicine.tw.
8. Teleconsultation.tw.
9. Teleconsultations.tw.
10. Telephone.tw.
11. ehealth.tw.
12. mhealth.tw.
13. ecare.tw.
14. e-care.tw.
15. Web-based.tw.
16. Remote.tw.

17. Remotely.tw.

18. Online.tw.

19. 1 or 2 or 3 or 4 or 5 or 6 or 7 or 8 or 9 or 10 or 11 or 12 or 13 or 14 or 15

20. 16 or 17 or 18

21. Consult.tw.

22. Consultation.tw.

23. Delivered.tw.

24. Delivery.tw.

25. Counseling.tw.

26. 21 or 22 or 23 or 24 or 25

27. 20 and 26

28. exp Diabetes Mellitus/

29. exp Obesity/ or exp Overweight/

30. exp Cardiovascular Diseases/

31. exp Kidney Diseases/

32. exp Renal Dialysis/

33. Diabetes.tw.

34. Overweight.tw.

35. Obesity.tw.

36. Cardiovascular disease.tw.

37. Heart disease.tw.

38. CVD.tw.

39. Coronary disease.tw.

40. CHD.tw.

41. Stroke.tw.

42. kidney disease.tw.

43. renal disease.tw.

44. Hypertension.tw.

45. Blood pressure.tw.

46. CKD.tw.

47. ESRD.tw.

48. ESKD.tw.

49. Dialysis.tw.

50. Hemodialysis.tw.

51. 28 or 29 or 30 or 31 or 32 or 33 or 34 or 35 or 36 or 37 or 38 or 39 or 40 or 41 or 42 or 43 or 44 or 45 or 46 or 47 or 48 or 49 or 50

52. exp Diet/

53. diet.tw.

54. dietary.tw.

55. food.tw.

56. Nutrition.tw.

57. Nutritional.tw.

58. Vegetable.tw.

59. fruit.tw.

60. fat.tw.

61. calories.tw.

62. 52 or 53 or 54 or 55 or 56 or 57 or 58 or 59 or 60 or 61

63. Adhered.tw.

64. Adherence.tw.

65. Comply.tw.

66. Complied.tw.

67. Compliance.tw.

68. alter.tw.

69. altered.tw.

70. modify.tw.

71. Satisfied.tw.

72. Satisfaction.tw.

73. Improve.tw.

74. Improvement.tw.

75. monitor.tw.

76. monitored.tw.

77. monitoring.tw.

78. reduce.tw.

79. reduction.tw.

80. reducing.tw.

81. 63 or 64 or 65 or 66 or 67 or 68 or 69 or 70 or 71 or 72 or 73 or 74 or 75 or 76 or 77 or 78 or 79 or 80

82. 19 or 27

83. 51 and 62 and 81 and 82

5 **Embase**

1. 'telecommunications'/exp OR telecommunications:ab,ti
2. 'internet'/exp OR internet:ab,ti
3. 'computers'/exp OR computers:ab,ti
4. Telecommunications:ab,ti
5. Internet:ab,ti
6. Telehealth:ab,ti
7. Telemedicine:ab,ti
8. Teleconsultation:ab,ti
9. Teleconsultations:ab,ti
10. Telephone:ab,ti
11. ehealth:ab,ti
12. mhealth:ab,ti
13. ecare:ab,ti
14. e-care:ab,ti
15. Web-based:ab,ti
16. Remote:ab,ti
17. Remotely:ab,ti

18. Online:ab,ti

19. 1 or 2 or 3 or 4 or 5 or 6 or 7 or 8 or 9 or 10 or 11 or 12 or 13 or 14 or 15

20. 16 or 17 or 18

21. Consult:ab,ti

22. Consultation:ab,ti

23. Delivered:ab,ti

24. Delivery:ab,ti

25. Counseling:ab,ti

26. 21 or 22 or 23 or 24 or 25

27. 20 and 26

28. 'diabetes'/exp OR (diabetes AND mellitus:ab,ti)

29. 'overweight'/exp OR overweight:ab,ti

30. 'obesity'/exp OR obesity:ab,ti

31. cardiovascular AND ('diseases'/exp OR diseases)

32. 'kidney'/exp OR kidney AND ('diseases'/exp OR diseases)

33. renal AND ('disease'/exp OR disease)

34. Diabetes:ab,ti

35. Overweight:ab,ti

36. Obesity:ab,ti

37. Cardiovascular disease:ab,ti

38. Heart disease:ab,ti

39. CVD:ab,ti

40. Coronary disease:ab,ti

41. CHD:ab,ti

42. Stroke:ab,ti

43. kidney disease:ab,ti

44. renal disease:ab,ti

45. Hypertension:ab,ti

46. Blood pressure:ab,ti

47. CKD:ab,ti

48. ESRD:ab,ti

49. ESKD:ab,ti

50. Dialysis:ab,ti

51. Hemodialysis:ab,ti

52. 28 or 29 or 30 or 31 or 32 or 33 or 34 or 35 or 36 or 37 or 38 or 39 or 40 or 41 or 42 or 43 or 44 or 45 or 46 or 47 or 48 or 49 or 50 or 51

53. 'diet'/exp OR diet:ab,ti

54. diet:ab,ti

55. dietary:ab,ti

56. food:ab,ti

57. Nutrition:ab,ti

58. Nutritional:ab,ti

59. Vegetable:ab,ti

60. fruit:ab,ti

61. fat:ab,ti

62. calories:ab,ti

63. 53 or 54 or 55 or 56 or 57 or 58 or 59 or 60 or 61 or 62

64. Adhered:ab,ti

65. Adherence:ab,ti

66. Comply:ab,ti

67. Complied:ab,ti

68. Compliance:ab,ti

69. alter:ab,ti

70. altered:ab,ti

71. modify:ab,ti
72. Satisfied:ab,ti
73. Satisfaction:ab,ti
74. Improve:ab,ti
75. Improvement:ab,ti
76. monitor:ab,ti
77. monitored:ab,ti
78. monitoring:ab,ti
79. reduce:ab,ti
80. reduction:ab,ti
81. reducing:ab,ti
82. 64 or 65 or 66 or 67 or 68 or 69 or 70 or 71 or 72 or 73 or 74 or 75 or 76 or 77 or 78 or 79 or 80 or 81
83. 19 or 27
84. 52 and 63 and 82 and 83

CINAHL (via EBSCOhost)

S19 S6 AND S13 AND S17 AND S18

S18 TI (Adhered OR Adherence OR Comply OR Complied OR Compliance OR Alter OR Altered OR Modify OR Modified OR Satisfied OR Satisfaction OR Improve OR Improves OR Improved OR Improvement OR Improvements OR Monitor OR Monitored OR Monitoring OR Reduce OR Reduction OR Reducing) OR AB (Adhered OR Adherence OR Comply OR Complied OR Compliance OR Alter OR Altered OR Modify OR Modified OR Satisfied OR Satisfaction OR Improve OR Improves OR Improved OR Improvement OR Improvements OR Monitor OR Monitored OR Monitoring OR Reduce OR Reduction OR Reducing)

S17 S14 OR S15 OR S16

S16 Diet OR Diets OR Dietary OR Food OR Nutrition OR Nutritional OR Vegetable OR Vegetables OR Fruit OR Fruits OR Fat OR Fats OR Calories

S15 (MH "Nutrition+")

S14 (MH "Diet+")

S13 S7 OR S8 OR S9 OR S10 OR S11 OR S12

S12 Diabetes OR Overweight OR Obesity OR "Cardiovascular disease" OR "Cardiovascular diseases" OR "Heart disease" OR "Heart diseases" OR CVD OR "Coronary disease" OR "Coronary diseases" OR CHD OR Strokes OR Stroke OR "kidney disease" OR "kidney diseases" OR "Renal disease" OR "Renal diseases" OR Hypertension OR "Blood pressure" OR CKD OR ESRD OR Dialysis OR Hemodialysis

S11 (MH "Dialysis+")

S10 (MH "Kidney Diseases+")

S9 (MH "Cardiovascular Diseases+")

S8 (MH "Obesity+")

S7 (MH "Diabetes Mellitus+")

S6 S1 OR S2 OR S3 OR S4 OR S5

S5 (Remote OR Remotely OR Online) AND (Consult OR Consultation OR Delivered OR Delivery OR Counseling)

S4 Telecommunications OR Internet OR Telehealth OR Telemedicine OR Teleconsultation OR Teleconsultations OR Telephone OR ehealth OR mhealth OR ecare OR e-care OR Web-based

S3 (MH "Computers and Computerization+")

S2 (MH "Internet+")

S1 (MH "Telecommunications+")
